# Supplementary material for: Hyperbolic Graph Embedding of MEG Brain Networks to Study Brain Alterations in Individuals With Subjective Cognitive Decline
Source: IEEE J Biomed Health Inform. Author manuscript; Available in PMC 2025 Mar 4. (PMC11700499; doi:10.1109/JBHI.2024.3416890)
Supplement: supp1-3416890 [file NIHMS2040372-supplement-supp1-3416890.pdf]

Supplementary material: Hyperbolic graph embedding of MEG brain networks to study brain alterations in individuals with subjective cognitive decline

I. Assignment of brain regions to subnetworks

Atlases delineating regions of interest (ROIs) within brain subnetworks deviate significantly from structural atlases like the AAL because they are based on functional patterns of communication instead of physical structures in the brain. To assign brain regions to distinct subnetworks, we conducted a comprehensive evaluation of various subnetwork studies [1]–[4]. We considered eight distinct brain subnetworks: posterior default mode network (pDMN), anterior default mode network (aDMN), dorsal attention network (DAN), frontoparietal network (FPN), visual network (VN), ventral attention network (VAN), salience network (SN), and sensorimotor network (SMN). We then devised a procedure that considered the spatial proximity between each AAL anatomical region and the central coordinates of different functional ROIs.

Each brain subnetwork contained anywhere between 2 to 14 functional ROIs. To represent each functional ROI, we used its 3D center coordinates. In order to assign AAL anatomical ROIs to these subnetwork functional ROIs, we employed a straightforward technique that considered the spatial proximity between these regions. Specifically, for each source A_k located within an anatomical ROI, we computed the distance $\mathrm{dist}(C_{A_k}, C_{F_j})$ to the center of the corresponding functional ROI C_{F_j}. We then defined an inclusion score, denoting the percentage of sources for which the distance fell within a defined threshold r, as follows:

$$P_{A,F} = \frac{1}{N_A} \sum_{N_A} \sum_{N_F} \begin{cases} 1, & \text{if } \mathrm{dist}(C_{A_k}, C_{F_j}) < r \\ 0, & \text{otherwise} \end{cases} \quad (1)$$

where A, N_A, and A_k are an ROI in AAL, the number of sources in A and the kth source in A. F is a subnetwork, F_j is the jth functional ROI belonging to F, N_F is the number of functional ROIs in F, $P_{A,F}$ is the percentage score of A belonging to F. Note that an average is only taken over the number N_A, not N_F, because the score calculates the percentage of *all* nodes in A that are near *any* ROI in F.

The distance threshold r was set to 1.5 cm. Last, the inclusion threshold t was selected such that A is assigned to F if $P_{A,F} > t$. Anatomical ROIs were allowed to be assigned to multiple subnetworks. The value of t was set at 0.3, as it limited all subnetworks to a reasonable while ensuring that regions known to belong to specific subnetworks were correctly assigned.

The list of anatomical ROIs belonging to each subnetwork are detailed below. Note that these networks exhibit hemispheric symmetry, encompassing both the right and left regions. For instance, the pDMN encompasses a total of 10 ROIs, with five in each hemisphere.

Posterior default mode network (pDMN): cingulate gyrus - posterior part; hippocampus, parahippocampus, angular gyrus, precuneus.

Anterior default mode network (aDMN): superior frontal gyrus - medial; superior frontal gyrus - medial orbital, cingulate gyrus - anterior part.

Dorsal attention network (DAN): precentral gyrus, inferior parietal gyrus, superior parietal gyrus.

Fronto-parietal network (FPN): inferior frontal gyrus - triangular, superior occipital lobe, inferior parietal gyrus, supramarginal gyrus, angular gyrus.

Visual network (VN): calcarine fissure, cuneus, lingual gyrus, middle occipital lobe, inferior occipital lobe.

Ventral attention network (VAN): precentral gyrus, inferior frontal gyrus - opercular, inferior frontal gyrus - triangular, insula, supramarginal gyrus, angular gyrus, middle temporal gyrus.

Salience network (SN): inferior frontal gyrus - orbital, olfactory cortex, cingulate gyrus - anterior part, hippocampus, parahippocampus, amygdala.

Sensori-motor network (SMN): supplementary motor area.

References

[1] W. Shirer, “Decoding subject-driven cognitive states with whole-brain connectivity patterns.” *Cerebral Cortex*, 2011.

[2] P. Boveroux, “Breakdown of within- and between-network resting state functional magnetic resonance imaging connectivity during propofol-induced loss of consciousness.” *Anesthesiology*, 2010.

[3] B. He, “Breakdown of functional connectivity in frontoparietal networks underlies behavioral deficits in spatial neglect.” *Neuron*, 2007.

[4] A. Janes, “Insula–dorsal anterior cingulate cortex coupling is associated with enhanced brain reactivity to smoking cues.” *Neuropsychopharmacology*, 2010.
